# Interplay of Physical, Psychological, and Social Frailty among Community-Dwelling Older Adults in Five European Countries: A Longitudinal Study

**DOI:** 10.1007/s11524-024-00831-5

**Published:** 2024-06-24

**Authors:** Lizhen Ye, Amy van Grieken, Tamara Alhambra-Borrás, Shuang Zhou, Gary Clough, Athina Markaki, Lovorka Bilajac, Hein Raat

**Affiliations:** 1 Department of Public Health, Erasmus MC, University Medical Center Rotterdam, P.O. Box 2040, 3000 CA Rotterdam, The Netherlands; 2 Polibienestar Research Institute - Universitat de València ES, Valencia, Spain; 3 Department of Maternal and Child Health, School of Public Health, Peking University, Beijing, China; 4 Department of Public Health, The University of Manchester, Manchester, UK; 5 Alliance for Integrated Care, Athens, Greece; 6 Faculty of Medicine, University of Rijeka, Rijeka, Croatia

**Keywords:** Bi-directional association, Cross-lagged panel modeling, Older adults, Physical frailty, Psychological frailty, Personalized prevention and care, Social frailty, Unidirectional association

## Abstract

Frailty is a dynamic condition encompassing physical, psychological, and social domains. While certain factors are associated with overall or specific frailty domains, research on the correlations between physical, psychological, and social frailty is lacking. This study aims to investigate the associations between physical, psychological, and social frailty in European older adults. The study involved 1781 older adults from the Urban Health Centres Europe project. Baseline and 1-year follow-up data were collected on physical, psychological, and social frailty, along with covariates. Linear regression analyzed unidirectional associations, while cross-lagged panel modeling assessed bi-directional associations. Participants’ mean age was 79.57 years (SD = 5.54) and over half were female (61.0%). Physical and psychological frailty showed bi-directional association (effect of physical frailty at baseline on psychological frailty at follow-up: *β* = 0.14, 95%CI 0.09, 0.19; reversed direction: *β* = 0.05, 95%CI 0.01, 0.09). Higher physical frailty correlated with increased social frailty (*β* = 0.05, 95%CI 0.01, 0.68), but no association was found between social and psychological frailty. This longitudinal study found a reciprocal relationship between physical and psychological frailty in older adults. A relatively higher level of physical frailty was associated with a higher level of social frailty. There was no association between social and psychological frailty. These findings underscore the multifaceted interplay between various domains of frailty. Public health professionals should recognize the implications of these interconnections while crafting personalized prevention and care strategies. Further research is needed to confirm these findings and investigate underlying mechanisms.

**Supplementary Information:**

The online version contains supplementary material available at 10.1007/s11524-024-00831-5.

## Introduction

Given the increasing population of older adults worldwide in the past two decades, frailty has become an emerging global health burden [1]. It affects individuals, families, caregivers, and health and social care systems negatively with its resulting negative health outcomes [2]. Frailty is potentially preventable [2], and it is defined as an increased state of vulnerability across multiple physiological systems when facing stressors [3]. It is dynamic, multidimensional, and often refers to the impact on the physical, psychological, and/or social domains [4].

Demographic characteristics (e.g., older age, female sex, lower education) and lifestyle behaviors (e.g., higher risk of alcohol use, less exercise) are associated with higher level of overall frailty or one of the separate domains of frailty (i.e., physical, psychological, and social) [5–7]. At the same time, some studies found that certain factors are associated with only specific domains of frailty [5–7]. For example, older age is associated with an increased level of physical frailty but not with psychological and social frailty [6], while female sex is associated with an increased level of physical and psychological frailty but not with social frailty [5]. A higher risk of daily alcohol use is associated with lower odds of greater social frailty level but not physical and psychological frailty [7]. Moreover, some studies have found that people can be frail in one or multiple domains concurrently (i.e., physical, psychological, and social) [8, 9], while some individuals are overall non-frail but frail in a specific domain [5].

However, previous studies have not explored the correlations between the three domains of frailty. Most previous studies have either explored the associations between different factors and separate domains of frailty or the association between frailty and specific diseases and conditions, mostly with regard to physical frailty [10, 11]. A comprehensive understanding of frailty development across these domains is crucial for preventing frailty in community-dwelling older adults. Currently, there is a gap in research on the connections between physical, psychological, and social frailty. Therefore, it is imperative to investigate how these domains of frailty interconnect over time to develop personalized interventions for promoting healthy aging and preventing frailty. By paying attention to these domains and their associations, we can develop effective interventions and address the nuanced needs of this vulnerable demographic, fostering enhanced health outcomes among older adults.

In this study, we assessed the bi-directional longitudinal associations between physical and psychological frailty, between physical and social frailty, as well as between psychological and social frailty. This was conducted in a large population-based sample of community-dwelling older adults in five European countries, with a 12-month follow-up.

## Methods

### Data Source

We conducted a longitudinal study using data from the Urban Health Centres Europe project, which aimed to improve the healthy aging of older adults in five European countries (the UK, Greece, Croatia, The Netherlands, Spain). The study conducted baseline measurements of participants in May 2015 and followed up with a second assessment 12 months later in June 2017 [12]. The project provided integrated care pathways and assessments for participants in the intervention group [13], which involved risk assessment, shared decision-making, and referral to care pathways aimed at preventing frailty, fall risk, loneliness, and inappropriate medication use. Face-to-face self-reported semi-structured interviews were conducted by a trained researcher at baseline and 1-year follow-up [13]. Ethical review procedures have been followed in all cities and approvals have been provided. Written informed consent was obtained from all participants. The study was registered as ISRCTN52788952 [12, 13]. Further details on the study design were described elsewhere [12, 13].

### Participants

**Fig. 1:**
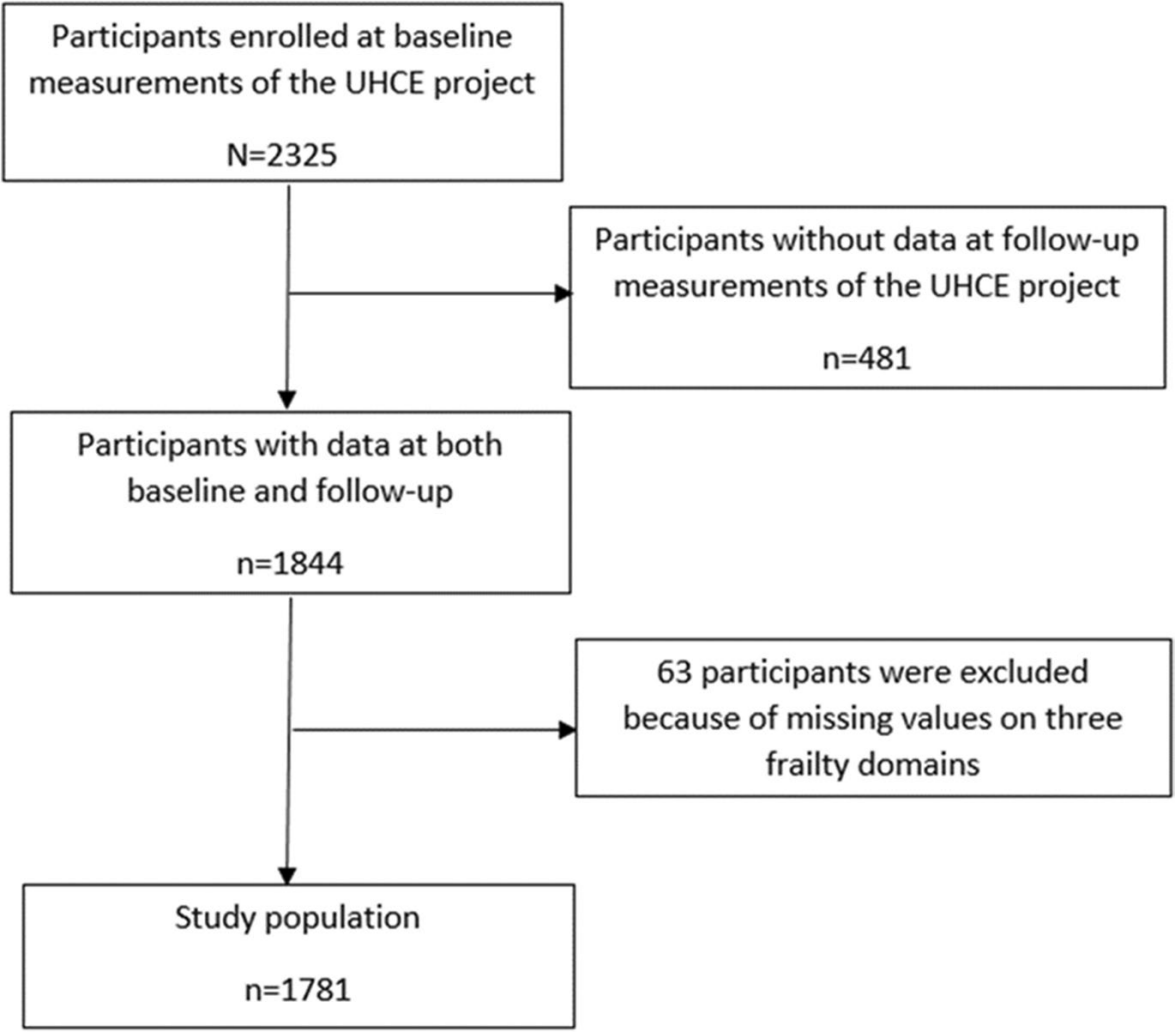
The flow chart of the study population

A total of 2325 older adults who lived independently and, according to their physician, could participate for at least 6 months were enrolled at baseline [12]. Participants who dropped out at follow-up (*n* = 481), with missing data on physical, psychological, and social frailty (*n* = 63) were excluded. Thus, 1781 participants were included in this study (Fig. 1).

### Procedures

#### Three Domains of Frailty

The Urban Health Centres Europe assessment evaluated frailty using the Tilburg Frailty Indicator, a validated tool consisting of 15 self-reported questions covering physical, psychological, and social domains [14–16]. Physical frailty is assessed by eight items covering physical health, unexplained weight loss, difficulties in walking, balance, hand strength, physical tiredness, eyesight, and hearing impairments. Psychological frailty is assessed by four items addressing problems with memory, feeling down, feeling nervous or anxious, and inability to cope with problems. Social frailty is assessed by three items regarding living alone, lack of social relationships, and lack of social support. The score range of physical frailty is 0 to 8, psychological is 0 to 4, and social is 0 to 3. Higher scores indicate greater frailty in each domain, with cutoff points of 3 for physical frailty, 2 for psychological frailty, and 2 for social frailty [17].

#### Covariates

The study’s covariates were assessed at baseline and included age (years), sex (female, male), education level (primary or less, secondary or equivalent, and tertiary or higher), country of residence (UK, Greece, Croatia, The Netherlands, and Spain), living composition (living with others/living alone), alcohol use (yes, no), physical activity (once a week or less/more than once a week), and the number of chronic conditions.

Educational level was classified into three categories regarding the highest level of education gained by a participant [18]. Alcohol use was assessed using the Alcohol Use Disorders Identification Test and dichotomized as a hazardous drinker or active alcohol use disorder (yes/no) [19]. Physical activity was measured by the frequency of low- or medium-energy-level activities [20]. Answers include “once a week or less” and “more than once a week” [5]. The number of chronic conditions was measured as a score (0 to 15) as having or not experienced 14 common chronic conditions and other conditions which did not mention (Supplementary Table S1). Covariates included whether the participants were in the intervention group (yes/no) and the frailty domain that was not the main outcome.

#### Statistical Analyses

The study population characteristics were described using descriptive statistics: continuous variables were summarized by means and standard deviation (SD) and categorical variables using frequencies and percentages.

Linear regression analyses were used to examine unidirectional associations for each outcome, with three models conducted: (1) a crude model adjusted for the intervention group, (2) a model adjusted for covariates and intervention group, and (3) a model additionally adjusted for the baseline assessment of the outcome. Given that “living composition” is one of the items of social frailty, we choose not to include it as a covariate in the models for social frailty as a primary outcome. “Betas (β)” reported in the results section are “standardized linear regression coefficients.” Standardized linear regression coefficients represent the strength and direction of the relationship between predictor variables and the outcome variable after standardizing all variables involved. Missing data was imputed five times using full conditional specification and pooled using Rubin rules.

Bi-directional associations between the frailty domains were investigated by a cross-lagged panel modeling approach using two-time measurements [21, 22]. This approach aims to assess the directional effects of one variable on another at different points in time [23]. “Betas (β)” reported in the results section of the cross-lagged panel model are “standardized linear regression coefficients.” These coefficients provide insights into how changes in one variable at an earlier time point are associated with changes in another variable at a later time point, while accounting for the variability and scales of the variables. Wald tests were performed to determine differences between opposing coefficients of lagged effects. While the cross-lagged panel modeling approach enables the analysis of interdependent variables’ reciprocal and longitudinal relationships under the assumption of synchronous measurements and stationary relationships [21–23]. However, the results should be interpreted with caution since these assumptions may be invalidated due to the data collection complexity and varying degrees of stationarity [21, 22]. The cross-lagged panel modeling accounted for the cross-sectional associations and continuity within repeated assessments over time, with covariates assessed at baseline and regressed on the three domains of frailty at 1-year follow-up. Given that “living composition” is one of the items of social frailty, we choose not to include it as a covariate in the models for social frailty as a primary outcome. The parameters in the cross-lagged panel modeling were estimated using the maximum likelihood estimation with robust standard errors to account for the non-normality of the data. In addition, full information maximum likelihood was used to account for missing values of the covariates [21].

Descriptive and unidirectional analyses (linear regressions) were conducted in the IBM SPSS Statistics for Windows (version 25 Armonk, NY, USA: IBM Corp). The cross-lagged panel modeling was performed with the lavaan package in R (version 4.1.2; R Development Core Team) within the RStudio (version 2021.09.2 × 64 ENG) platform.

#### Non-response Analysis

The response group (*n* = 1844) included participants with both baseline and follow-up information, while the non-response group (*n* = 481) dropped out at follow-up. Baseline characteristics were compared between groups using *T*-tests for continuous variables and chi-square tests for categorical variables.

## Results

### Participants Characteristics

Table 1 presents the descriptive statistics on all the covariates. Participants (*n* = 1781) had a mean age of 79.57 years (SD = 5.54), and most were female (61.0%) from the UK (25.4%), Croatia (23.8%), and Spain (22.2%). Over half completed secondary education (64.0%) or lived with others (61.1%). The majority used no alcohol (68.8%) and exercised more than once a week (72.7%). Participants’ mean number of chronic conditions is 3.9 (SD = 1.89). The results in Supplementary Table S2 show that among the participants with physical frailty, more than half of them (53.4%) experienced psychological frailty and 36.2% of them experienced social frailty. Among the participants who experienced social frailty, 53.2% of them were psychologically frail.

**Table 1:** Participants’ characteristics (*n* = 1781)

Characteristic	*n* (%)
Age, mean ± SD	79.6 ± 5.5
Sex, % of female	1087 (61.0%)
Country	
The Netherlands	268 (15.0%)
Greece	243 (13.6%)
Croatia	423 (23.8%)
Spain	395 (22.2%)
The UK	452 (25.4%)
Educational level	
Primary or less	440 (24.7%)
Secondary	1140 (64.0%)
Tertiary or higher	177 (9.9%)
Household composition	
Living with others	1088 (61.1%)
Living alone	688 (38.6%)
Alcohol use	
Yes	458 (25.7%)
No	1226 (68.8%)
Exercise	
More than once a week	1295 (72.7%)
Once a week or less	474 (26.6%)
The number of chronic conditions	3.9 ± 1.9

Note: The table is based on a non-imputed dataset

Missing items: age, 1; educational level, 24; household composition, 5; alcohol use, 97; exercise, 12; the number of chronic conditions, 1

### The Unidirectional Associations

Tables 2, 3, and 4 present unidirectional associations between the physical, psychological, and social frailty domains. Our results in Table 2 show that a higher physical frailty score at follow-up was associated with a relatively higher psychological frailty score (*β* = 0.41, 95%CI 0.32,0.50) after adjusting for covariates. However, for social frailty, the association turned to non-significance when adjusting for covariates and baseline physical frailty score. The results in Table 3 show that a higher psychological frailty score at follow-up was associated with a relatively higher physical (*β* = 0.15, 95%CI 0.12,0.18) and social (*β* = 0.11, 95%CI 0.05,0.16) frailty score after adjusting for covariates. However, for social frailty at baseline, the association attenuated to non-significance when accounting for baseline psychological frailty. In Table 4, the results show that a higher social frailty score at follow-up was associated with a relatively higher physical (*β* = 0.04, 95%CI 0.02,0.06) and psychological frailty score (*β* = 0.12, 95%CI 0.08,0.16) after accounting for covariates. However, for a psychological frailty score at baseline, the association abated to non-significance when accounting for social frailty at baseline.

**Table 2:** Unidirectional association between physical frailty at follow-up and baseline psychological and social frailty (*n* = 1781)

Model	β (95%CI) related to psychological frailty at baseline^a^	β (95%CI) related to social frailty at baseline^a^
Adjusted for the intervention group	0.75 (0.66, 0.85)*	0.49 (0.38, 0.60)*
Adjusted for covariates and intervention group	0.41 (0.32, 0.51)*^b^	0.05 (−0.06, 0.15)^c^
Additionally adjusted for physical frailty at baseline	0.10 (0.02, 0.18)*	−0.02 (−0.11, 0.07)

The table is based on an imputed dataset. Values represent standardized linear regression coefficients (*β*, 95% confidence intervals)

^*^Represents *P* < 0.05

^a^Effect estimates are unstandardized linear regression coefficients and 95% confidence intervals

^b^Adjusted for the intervention group, age, sex, country, education level, household composition, alcohol use, exercise, the number of chronic conditions, and social frailty at baseline

^c^Adjusted for the intervention group, age, sex, country, education level, alcohol use, exercise, the number of chronic conditions, and psychological frailty at baseline

**Table 3:** Unidirectional association between psychological frailty at follow-up and baseline physical and social frailty (*n* = 1781)

Model	*β* (95%CI) related to physical frailty at baseline^a^	*β* (95%CI) related to social frailty at baseline^a^
Adjusted for the intervention group	0.22 (0.19, 0.24)*	0.27 (0.21, 0.32)*
Adjusted for covariates and intervention group	0.15 (0.12, 0.18)*^b^	0.11 (0.05, 0.16)*^c^
Additionally adjusted for psychological frailty at baseline	0.08 (0.05, 0.10)*	−0.02 (−0.11, 0.07)

The table is based on an imputed dataset. Values represent standardized linear regression coefficients (*β*, 95% confidence intervals)

^*^Represents *P* < 0.05

^a^Effect estimates are unstandardized linear regression coefficients and 95% confidence intervals

^b^Adjusted for social frailty, intervention group, age, sex, country, education level, exercise, and the number of chronic conditions

^c^Adjusted for physical frailty, intervention group, age, sex, country, education level, exercise, and the number of chronic conditions

**Table 4:** Unidirectional association between social frailty at follow-up and baseline physical and psychological frailty (*n* = 1781)

Model	*β* (95%CI) related to physical frailty at baseline^a^	*β* (95%CI) related to psychological frailty at baseline^a^
Adjusted for the intervention group	0.11 (0.09, 0.13)*	0.21 (0.17, 0.25)*
Adjusted for covariates and intervention group	0.04 (0.02, 0.06)*^b^	0.12 (0.08, 0.16)*^c^
Additionally adjusted for social frailty at baseline	0.02 (0.00, 0.04)*	0.01 (−0.02, 0.04)

The table is based on an imputed dataset. Values represent standardized linear regression coefficients (*β*, 95% confidence intervals)

^*^Represents *P* < 0.05

^a^Effect estimates are unstandardized linear regression coefficients and 95% confidence intervals

^b^Adjusted for psychological frailty, intervention group, age, sex, country, education level, exercise, and the number of chronic conditions

^c^Adjusted for physical frailty, intervention group, age, sex, country, education level, exercise, and the number of chronic conditions

### The Bidirectional Associations

The bi-directional associations between the three frailty domains (physical, psychological, and social) are presented in Supplementary Figure S1 to S3.

For physical and psychological frailty (Supplementary Figure S1), the autoregressive coefficients (physical frailty *β* = 0.58, 95%CI 0.54, 0.62 and psychological frailty *β* = 0.45, 95%CI 0.41, 0.49) showed moderate stability over 1 year, which indicated both physical and psychological frailty at follow-up had moderate influence from the baseline. In other words, these *β* coefficients can be understood as residual correlations, akin to how much of the follow-up frailty level can be attributed to its baseline level. The result of the lagged effects was comparable with the reported unidirectional associations (Tables 2 and 3). The cross-lagged effect (*β*) from physical frailty at baseline on psychological frailty at follow-up is 0.14 (95%CI 0.09, 0.19), suggesting that a higher physical frailty score at baseline was associated with a relatively higher psychological frailty score at follow-up. Conversely, a higher psychological frailty score at baseline was associated with a relatively higher physical frailty score at follow-up (*β* = 0.05, 95%CI 0.01, 0.09). The lagged effects for both paths were statistically significant, with the path from physical frailty at baseline to psychological frailty at follow-up being stronger than the reversed path (Wald test for comparing lagged effects: *P* < 0.05).

For physical and social frailty (Supplementary Figure S2), the autoregressive coefficients (physical frailty *β* = 0.59, 95%CI 0.55, 0.63 and social frailty *β* = 0.65, 95%CI 0.62, 0.68) showed moderate stability over 1 year, which indicated both physical and social frailty at follow-up had moderate influence from the baseline. The result of the lagged effects was comparable with the reported unidirectional associations (Tables 2 and 4). A higher physical frailty score at baseline was associated with a relatively higher social frailty score at follow-up (*β* = 0.05, 95%CI 0.01, 0.68), while no association was observed in the reversed direction.

For social and psychological frailty (Supplementary Figure S3), the autoregressive coefficients (social frailty *β* = 0.65, 95%CI 0.62, 0.68 and psychological frailty *β* = 0.47, 95%CI 0.43, 0.51) showed moderate stability over 1 year, which indicated both social and psychological frailty at follow-up had moderate influence from the baseline. The result of the lagged effects was comparable with the reported unidirectional associations (Tables 3 and 4). No associations were observed in both directions.

Compared with the response group (*n* = 1844), the non-response group (*n* = 481) was older (80.5 ± 5.89 years), had higher baseline physical (3.23 ± 2.21) and psychological (1.32 ± 1.14) frailty scores, and had lower education levels. They were also more likely to come from the NL or Greece and to exercise once a week or less (all *P* < 0.05). No other statistically significant differences were found.

## Discussion

We studied longitudinal associations between physical, psychological, and social frailty in community-dwelling older adults across five European countries. Results show a bi-directional association between physical and psychological frailty, with a stronger effect from physical to psychological frailty. Physical frailty at baseline was associated with social frailty at follow-up. No association was found between social and psychological frailty. These results were consistent across different modeling strategies.

The present study confirms firstly a bi-directional association between physical and psychological frailty. In line with previous studies [24, 25], our findings indicated that older adults with a relatively higher level of physical frailty may experience a higher level of psychological frailty over 1-year follow-up and vice versa. It might be that factors related to physical frailty, such as difficulties in walking or weaker hand strength, may contribute to psychological frailty. For example, Cooper R. et al. reported that people with physical difficulties were more likely to have lower levels of mental well-being a few years later, encompassing psychological and emotional health [26]. According to Ohrnberger J. et al., individuals’ mental well-being and their ability to access information on their health would affect their decision-making process and thus further affect their lifestyle choices such as physical activity [27]. This may explain the association between psychological frailty and physical frailty, i.e., the reversed direction. Factors that are related to psychological frailty among older people in this study, such as emotional status, the inability to cope with problems or memory problems, might affect physical frailty via health-related decision-making or behaviors [28]. Due to this “chain reaction,” their physical health might be affected as well [29]. Policies and interventions for older adults should consider their mental well-being alongside physical health. Integrating mental health screening and support with physical health assessments and interventions is crucial. Healthcare providers should be trained to recognize and address the psychological needs of older adults with physical frailty to improve their overall well-being. Additionally, the effect of physical frailty on psychological frailty was relatively stronger than psychological frailty on physical frailty in the current study. Studies on the mechanisms underlying the effect strength between physical and psychological frailty are needed.

Secondly, a unidirectional association between physical and social frailty was observed. In line with a previous study [11], older adults with higher level of physical frailty may experience relatively higher level of social frailty after 1 year. Factors related to physical frailty (e.g., poor physical health, difficulties in walking, and physical tiredness) could result in people spending less time outdoors [30]. This, in turn, might further lead to reduced social interactions and a decline in social aspects of life. However, unlike previous studies [11, 31–33], we did not observe a statistically significant effect from social frailty to physical frailty. This might be due to the limited range of social frailty indicators used in the Tilburg Frailty Indicator which includes only three items: living alone, lack of social relationships, and lack of social support. Thus, the variation in social frailty statuses is relatively limited, and differences in social frailty between individuals may be less pronounced in our study. Interventions targeting physical frailty may have positive effects on social well-being by improving physical health and mobility. This can be achieved through promoting access to community resources, creating age-friendly environments that support social participation, and providing opportunities for social interaction and support. Further studies are needed to explore the potentially reciprocal relationship between physical and social frailty. Additionally, different studies used various criteria to assess social frailty [5, 11, 31, 32]. To validate our findings, a shared standard assessment for social frailty is needed in further studies.

Thirdly, the present study did not find a statistically significant association between social and psychological frailty. This may suggest that different factors contribute to these two aspects of frailty, and interventions targeting one may not necessarily impact the other. Our finding contrasts with previous studies [31, 34, 35], which may be attributed to the use of different psychological and social frailty scales in those studies. For example, the perception of “feeling unhelpful to friends or family” is often considered a social variable but may be better classified as a psychological variable due to its inherent nature as a subjective emotion [31]. This distinction highlights the subtle challenges inherent in distinguishing psychological frailty from social frailty. The differences in associations reported in our study compared with other studies may be partially attributable to the inherent interconnectedness of these conceptual areas. Further research is needed to explore the pathways of associations between social and psychological frailty based on broader standard assessments of two frailty domains.

## Strengths and Limitations

The strengths of the current study are the large population-based sample and the repeated measurements of physical, psychological, and social frailty. We evaluated the longitudinal associations between different frailty domains, simultaneously considering potential temporal association in the reverse direction within the same time. Additionally, our methodological strategy on bidirectional associations accounted for the cross-sectional associations between different frailty domains and continuity within repeated assessments over a 1-year follow-up at the same time [21].

However, some limitations should be considered as well. First, while we employed a reliable and valid instrument, the TFI, to assess physical, psychological, and social frailty, there are still some considerations. According to Zhang et al. [15], future research exploring frailty should incorporate additional items in the psychological and social domains, such as feelings of insecurity and the number of social contacts [14]. This would require a more comprehensive instrument to further validate the results of our study. Second, it is imperative to acknowledge that the variables of alcohol use and physical activity in our study were reliant on self-reported data, potentially introducing recall bias. Consequently, the interpretation of our findings should be approached with caution. Third, considering the bi-directional association between physical and psychological frailty and the unidirectional association between physical and social frailty, physical frailty might be a mediator or moderator on other frailty domains. We could not explore this association due to the two waves of assessments in this study. Further studies with at least three waves of assessments are needed.

## Conclusions

This longitudinal study found a reciprocal relationship between physical and psychological frailty in older adults, with a relatively stronger effect from physical to psychological frailty than reversed direction. A relatively higher level of physical frailty was associated with a higher level of social frailty. There was no association between social and psychological frailty. These findings underscore the multifaceted interplay between various domains of frailty and have the potential to enhance the understanding of frailty and inform the development of holistic care strategies. Further research is needed to confirm these findings and investigate underlying mechanisms. Health practitioners and public health professionals should consider these associations when providing personalized prevention and care for older adults with frailty.

### Supplementary Information

Below is the link to the electronic supplementary material.

Supplementary file1 (DOCX 203 KB)

## Data Availability

The datasets analysed during the current study are not publicly available due to privacy/ethical restrictions but are available from the corresponding author on reasonable request.
