# Supplementary material for: Interplay of Physical, Psychological, and Social Frailty among Community-Dwelling Older Adults in Five European Countries: A Longitudinal Study
Source: J Urban Health. 2024 Jun 24;101(4):730–9. doi: 10.1007/s11524-024-00831-5 (PMC11329455; doi:10.1007/s11524-024-00831-5)
Supplement: Supplementary file 1 — Supplementary file1 (DOCX 203 KB) [file 11524_2024_831_MOESM1_ESM.docx]

**Supplementary materials**

**Supplementary Figure S1. Cross-lagged model of association between physical frailty and psychological frailty (n=1781).**

**
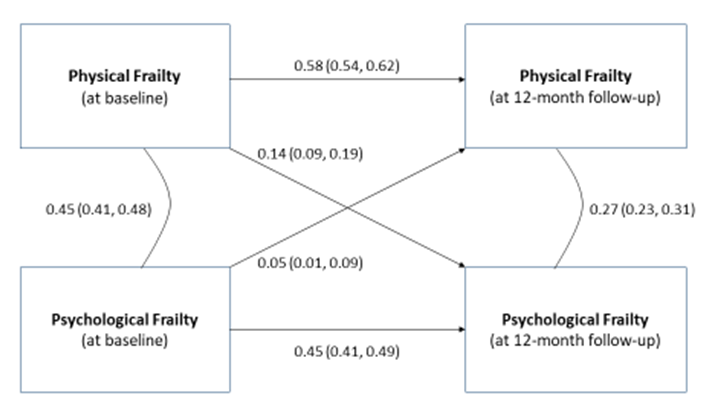
**

Values represent standardized linear regression coefficients (β, 95% confidence intervals). These coefficients represent the effect size in the model as well and considered baseline covariates, including intervention group, social frailty at baseline, age, sex, country, education level, exercise, household composition, alcohol use, the number of chronic conditions. Model fit: RMSEA=0.153, CFI=0.708 and TLI=0.348. Wald test comparing lagged pathways: *P*<0.05.

**Supplementary Figure S2. Cross-lagged model of association between physical frailty and social frailty (n=1781).**


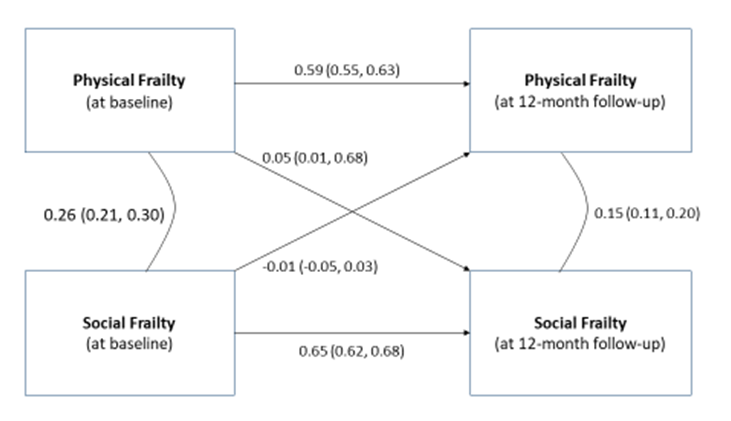


Values represent standardized linear regression coefficients (β, 95% confidence intervals). These coefficients represent the effect size in the model as well and considered baseline covariates, including intervention group, psychological frailty at baseline, age, sex, country, education level, exercise, alcohol use, the number of chronic conditions. Model fit: RMSEA=0.165, CFI=0.690 and TLI=0.303. Wald test comparing lagged pathways: P<0.05.

**Supplementary Figure S3. Cross-lagged model of association between social frailty and psychological frailty (n=1781).**


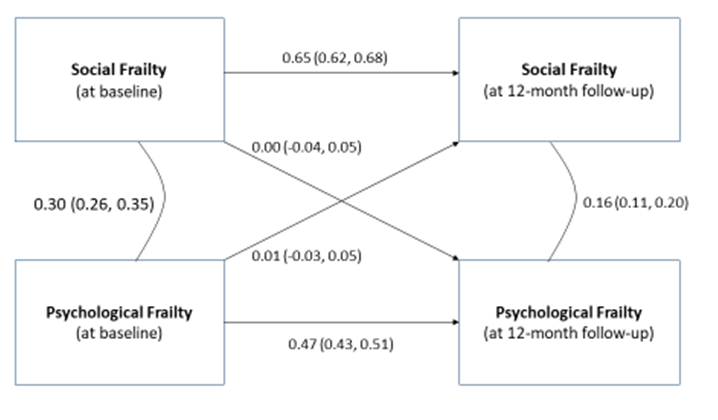


Values represent standardized linear regression coefficients (β, 95% confidence intervals). These coefficients represent the effect size in the model as well and considered baseline covariates, including intervention group, physical frailty at baseline, age, sex, country, education level, exercise, the number of chronic conditions. Model fit: RMSEA=0.133, CFI=0.763 and TLI=0.462. Wald test comparing lagged pathways: P=0.8.

**Supplementary Table S1. List on chronic conditions.**

| NO. | Has a doctor ever told you that you had one or more of these conditions? | Answer Options |
| --- | --- | --- |
| 1 | A heart attack including myocardial infarction or coronary thrombosis or any other heart problem including congestive heart failure | Yes/No |
| 2 | High blood pressure or hypertension | Yes/No |
| 3 | High blood cholesterol | Yes/No |
| 4 | A stroke or cerebral vascular disease | Yes/No |
| 5 | Diabetes or high blood sugar | Yes/No |
| 6 | Chronic lung disease such as chronic bronchitis or emphysema | Yes/No |
| 7 | Asthma | Yes/No |
| 8 | Arthritis, including osteoarthritis, or rheumatism | Yes/No |
| 9 | Osteoporosis | Yes/No |
| 10 | Cancer or malignant tumour, including leukaemia or lymphoma, but excluding minor skin cancers | Yes/No |
| 11 | Stomach or duodenal ulcer, peptic ulcer | Yes/No |
| 12 | Parkinson's disease | Yes/No |
| 13 | Cataract | Yes/No |
| 14 | Hip fracture or femoral fracture | Yes/No |
| 15 | Other conditions, not yet mentioned | Yes/No |

**Supplementary Table S2. Baseline distribution of physical, psychological, and social frailty (n=1781).**

|  | Psychological frailty | | | Social frailty | | |
| --- | --- | --- | --- | --- | --- | --- |
| Physical frailty | No | Yes | *P-value* | No | Yes | *P-value* |
| No | 667 (80.3%) | 164 (19.7%) | *P*<0.001 | 651 (78.3%) | 180 (21.7%) | *P*<0.001 |
| Yes | 443 (46.6%) | 507 (53.4%) |  | 606 (63.8%) | 344 (36.2%) |  |
| Social frailty |  |  |  |  |  |  |
| No | 865 (68.8%) | 392 (31.2%) | *P*<0.001 |  |  |  |
| Yes | 245 (46.8%) | 279 (53.2%) |  |  |  |  |
